# Altmetric Analysis of Dermatology Manuscript Dissemination During the COVID-19 Era: Cross-Sectional Study

**DOI:** 10.2196/46620

**Published:** 2023-08-16

**Authors:** Harrison Zhu, Vishnu Narayana, Kelvin Zhou, Anisha B Patel

**Affiliations:** 1 School of Medicine Baylor College of Medicine Houston, TX United States; 2 Department of Dermatology UT MD Anderson Cancer Center Houston, TX United States

**Keywords:** altmetric, media dissemination, citation number, bibliometric, attention score, social media

## Abstract

**Background:**

Alternative bibliometrics or altmetrics, is a measure of an academic article’s impact on social media outlets, which is quantified by the Altmetric Attention score (AAS). Given a lack of data for altmetric trends during the COVID-19 pandemic, we conducted a comprehensive, multivariable analysis of top dermatology manuscripts published during this time period.

**Objective:**

We aim to assess (1) the relationship between traditional bibiliometrics and Altmetrics and (2) factors associated with high AAS.

**Methods:**

All abstracted articles published in the top-5 (ranked by SCImago Journal Rankings) peer-reviewed dermatology journals published in 2021 were included in our study. We collected AAS as the dependent variable and categorical predictor variables included journal title, whether a conflict of interest existed, open access status, whether the article was related to COVID-19 or skin-of-color research, and the type of research (eg, clinical, basic science, review, etc). Numerical predictor variables consisted of the impact factor of journal, total citations, and number of authors. Multivariable linear or logistic regression models were used.

**Results:**

The relationship between AAS and citation number was significant by multivariable analysis during the COVID-19 pandemic (*P*<.001). Numerous factors, including studies related to COVID-19, whether the article was open access, title of the journal, and journal impact factor were also independently related to higher AAS (*P*<.002).

**Conclusions:**

Our results validate the use of altmetrics as a complement to traditional bibliometrics, especially in times of widespread scientific interest. Despite existing in a complex realm of bibliometrics, there are also discernable patterns associated with higher AAS. This is especially relevant in the era of growing technologic importance and utility to assess the impact of scientific works within the general public.

## Introduction

With rapid web-based communication, scientific literature is more widely disseminated now than ever [1]. Traditional metrics (eg, citation number), however, do not account for the dissemination of scientific research through general public engagement. Altmetric Attention score (AAS) is based on both the quality and quantity of mentions in media sources not considered by traditional bibliometrics and is calculated by a point system, with each attention source being assigned a specific weight (Table 1) [2-4]. While traditional bibliometrics may take time to progress, AAS tracks the immediate spread of scholarly work and assesses dissemination shortly after publication [1].

**Table 1:** Contributors to Altmetric Attention Score and weight of each source.

Attention source	Weight	Modifier notes
News outlet	8	News outlets ranked by tiers. More popular news outlets have greater contributions (eg, NY times>2Minute Medicine)
Blog	5	—^a^
Policy document	3	+3 per policy document mention
Patent	3	+3 per patent in “different” jurisdictions
Wikipedia	3	Mention in multiple pages does not increase the score more than single page mention
Peer review (Publons, Pubpeer)	1	—
F1000	1	—
Syllabi	1	—
Facebook	0.25	Only a curated list of public Pages count
Reddit	0.25	—
Stack Exchange	0.25	—
YouTube	0.25	—
Twitter	0.25-1.1	Score range based on following (retweets have a 0.85x modifier): Reach: increased with account follower number Promiscuity: decreased with frequency that account tweets about research output Bias: decreased with frequency that account tweets about research from the same domain (promotional intent)

^a^Not available.

There have been recent concerns related to inadequacies of solely relying on traditional bibliometrics to measure impact [5]. Thus, bibliometric research has been increasingly incorporating altmetric parameters. For example, a novel artificial intelligence bibliometric search engine model emphasized considering altmetric parameters as a strength of the model [6].

Fields including radiology, pathology, or orthopedic surgery, have shown strong relationships between AAS and traditional bibliometrics [7-9], representing a case for altmetrics to complement traditional bibliometrics [8]. In dermatology, however, evidence of this relationship is scarce [10].

The COVID-19 pandemic held unique ramifications for scientific article dissemination, resembling a sudden assault on an area of previously unknown vulnerability [11]. These novel circumstances made early identification of research outcomes crucial for guiding clinical decision-making, decreasing the effectiveness of traditional bibliometrics [11].

Given the novel circumstances, we suspected that there may be unique AAS trends in dermatology publications during the COVID-19 pandemic. With the increasing usage of social media in disseminating dermatologic information [12] and a scarcity of data on AAS trends in dermatology during the pandemic, we sought to perform a comprehensive Altmetric analysis to assess the following during this time period:

1. What is the nature of the relationship between AAS and citation number?
2. What factors are associated with a higher AAS score?

## Methods

All full-length, abstracted articles published in the top-5 peer-reviewed dermatology journals ranked by the SCImago Journal Rankings (SJR) from 2021 were included in our study. As a newer generation measure of journal impact, SJR has been deemed a more reliable measure of impact by accounting for factors such as dilutional citations (eg, editor self-citations) which can falsely inflate a journal’s impact factor (IF) [13].

For each journal, we reviewed every abstracted manuscript from January-December issues of 2021 and AAS scores were collected using the Altmetric Bookmarklet tool (Euan Adie) [14]. Other article-specific parameters were gathered as independent variables. The categorical independent variables included journal title, whether a conflict of interest existed, open access status, whether the article was related to COVID-19 or skin-of-color research, and the type of research (eg, clinical, basic science, review, etc). Numerical independent variables consisted of IF of journal, total citations, and number of authors.

All articles were accessed in a 2-week window in June 2022 to avoid variability in AAS changes.

Univariable linear regression or Kruskal-Wallis testing was used, and significant predictors of AAS were incorporated into a multivariable linear regression or quasi-Poisson logistic regression model, respectively. Calculations were done in RStudio (version 2022.02.0; Joseph J. Allaire).

## Results

**Figure 1:**
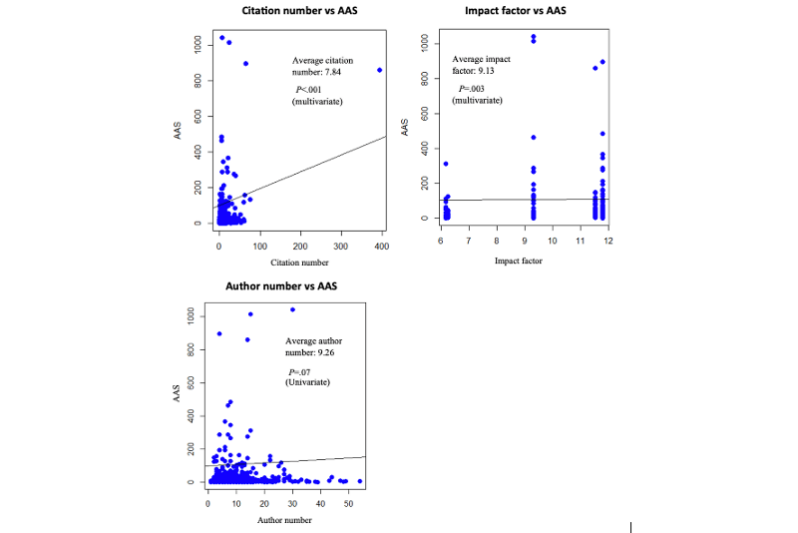
Relationships between AAS and quantitative manuscript factors. Plots of AAS against citation number, impact factor (IF), and author number with a univariable regression line. Both citation number and IFs were significant by univariable linear regression and incorporated into the multivariable regression model. Overall multivariable regression model: AAS=IF*3.43+Citation Number*2.08–25.24; Adjusted R2=0.2; *P*<.001. AAS: Altmetric Attention Score.

We analyzed 747 articles, with an average AAS of 24.2. Articles related to COVID-19 or skin-of-color research consisted of 3.6% (27/747) and 1.1% (8/747) of the total article population, respectively. Clinical research was the most popular article type (494/747, 66.1%; Table 2). In our multivariable logistic regression model, “JAMA dermatology” predicted higher AAS and “Journal of the European Academy of Dermatology and Venereology” predicted lower AAS (*P*<.001). The presence of conflicts of interest and open access articles predicted higher AAS (*P*<.001). COVID-19 research also predicted higher AAS (*P*<.001), despite accounting for only 4% (27/747) of total publications (Table 2). In our multivariable linear regression model, both citation number and IF independently correlated with AAS, accounting for 20% of the variation in AAS (*P*<.001; Figure 1).

**Table 2:** Relationships between Altmetric Attention Score (AAS) and categorical manuscript factors.

			Count (%)		Average Altmetric Attention Score		95% CI		Univariable *P* Value		Multivariable *P* Value
Total			747 (100)		24.2		18.3-30.2		—^a^		—
**Journal**											
	*British Journal of Dermatology* (BJD)	179 (24.0)		29.5		12.2-46.8		.27		—	
	*American Journal of Clinical Dermatology* (AJCD)	68 (9.1)		9.26		5.29-13.2		.32		—	
	*JAMA Dermatology* ^b^	107 (14.3)		66		44.5-87.5		<.001		<.001	
	*Journal of the American Academy of Dermatology* (JAAD)^b^	195 (26.1)		19.9		10.8-28.9		<.001		.72	
	*Journal of the European Academy of Dermatology and Venereology* (JEADV)^b^	198 (26.5)		6.31		2.79-9.83		<.001		<.001	
**Conflicts of interest^b^**											
	Yes	376 (50.3)		32.7		22.7-42.8		.002		.002	
	No	371 (49.7)		15.6		9.4-21.8		.002		.002	
**Open access^b^**											
	Yes	285 (38.2)		39.3		25.7-52.8		.001		<.001	
	No	462 (61.8)		15		10.3-19.6		.001		<.001	
**COVID-19–related?^b^**											
	Yes	27 (3.6)		194		60.5-327.5		<.001		<.001	
	No	720 (96.4)		17.9		14.7-21.0		<.001		<.001	
**Skin-of-color related?^b^**											
	Yes	8 (1.1)		87.2		–21.12 to 195.6		.01		.41	
	No	739 (98.9)		23.5		17.6-29.5		.01		.41	
**Article type**											
	Systematic Review	118 (15.8)		15.4		8.70-22.2		.58		—	
	Narrative Review	84 (11.2)		8.46		5.1-11.9		.06		—	
	Case report or series	14 (1.9)		93.9		–42.4 to 230.3		.11		—	
	Clinical^b^	494 (66.1)		28.4		13.1-26.0		.03		.34	
	Basic science^b^	31 (4.1)		5.55		3.3-7.8		.05		.15	
	Other	6 (0.8)		8.67		–7.6 to 25.0		.39		—	

^a^Not available.

^b^Indicates variables found significant by univariable Kruskal-Wallis testing that were incorporated into the multivariable logistic regression model.

## Discussion

The magnitude of the relationship between AAS and citation number is not well quantified [1], but our multivariable model (AAS=IF*3.43+Citation Number*2.08–25.24, *R*^2^=0.2, *P*<.001) quantifies the strength of this relationship.

Previous studies have analyzed the attributes of the top COVID-19 articles [15,16], but none have directly compared the AAS scores of COVID-19–related versus non–COVID-19–related articles. We found that despite consisting of only 4% (27/747) of the manuscripts published, the average AAS for COVID-19–related articles was nearly 10-fold higher than non–COVID-19–related articles, which was significant by multivariable analysis (*P*<.001). The COVID-19 pandemic represents the first international, widespread assault on human health in the modern era, resulting in a frantic race to understand and combat the novel coronavirus by scientists and laypeople alike. Thus, it is natural that the popularity of any manuscript related to COVID-19 would be reflected in both traditional and alternative bibliometrics.

Outside of studies related to COVID-19, we found that AAS was associated with citation numbers during this period. In dermatology, a previous study did not find a significant association between AAS and citation number [10], suggesting that the scientific interest sparked by the COVID-19 pandemic potentially strengthened the relationship between alternative and traditional bibliometrics. Given this close relationship, our findings support the potential for use of AAS complimentary to traditional bibliometrics especially in eras of global interest in scientific works.

Web of Science (WoS) is a database distinct from Scopus, the main database used in SJR. We used the Scopus or SJR database to determine the top-5 dermatology journals because journal rankings by discipline in WoS are not publicly published. Additionally, Scopus or SJR includes a broader spectrum of journals and provides a faster citation analysis [17]. Nonetheless, assessing the differences between altmetrics in top WoS versus Scopus or SJR rankings is an interesting direction for future research as both databases have unique attributes [17].

There are multiple reasons for the use of AAS. For researchers, altmetrics may be a clue for an urgent topic to investigate. For community members, the use of altmetrics promotes a form of patient-centered care, where the viewpoints of the general public are considered by investigators through the AAS. Greater consideration of altmetrics by current researchers in their scholarly pursuits may result in increased engagement with the future generation of researchers more likely to be on social media channels considered by the altmetric scoring system, thus inspiring young or aspiring researchers to focus on research topics particularly salient to society.

The profiles of the authors that publish in journals with higher AAS were outside the scope of our study as we focused on specific articles rather than authors. However, we found that the presence of conflicts of interest was independently related to higher AAS. We suspect that authors that publish in journals with greater AAS may have more funding or affiliations, resulting in an increased likelihood of conflicts of interest. In fact, AAS patterns seem to emerge early in training, as dermatologists from medical schools with greater research funding had significantly higher total AAS in their early research papers than those who did not (67.9 vs 22.9 *P*<.001) [18]. Other author-specific parameters, such as H-index may also be correlated to higher AAS publications.

Limitations of our analysis include the exclusion of nonabstracted articles, which may have different AAS patterns. It is also important to note that AAS should only complement and not replace other traditional metrics, as they are not a traditional marker of scientific importance and may further legitimize sensationalism if taken in isolation [1]. Nonetheless, we outline a model that demonstrates a significant relationship between AAS and traditional bibliometrics showing that there are several independent predictors of AAS of dermatology articles published during the COVID-19 pandemic.

The field of bibliometrics is undoubtedly complex, with multiple databases and methods to assess impact, and external forces, such as that of the COVID-19 pandemic, that can affect bibliometric patterns. Altmetrics are emerging as a unique tool in bibliometrics with utility that is especially relevant with the accessibility of technology in the modern era. There are multiple reasons to consider the public engagement of scientific manuscripts through alternative bibliometrics, including understanding of topics that are currently influential within the status quo, a focus on patient-centered care, and the potential to inspire future generations of scientists. While our work clarifies relationships between altmetrics and manuscript-specific factors such as citation number, further research is required to assess the relationship between author-specific, rather than manuscript-specific predictors of higher AAS.

## Abbreviations

AAS: Altmetric Attention Score
IF: impact factor
SJR: SCImago Journal Ranking
WoS: Web of Science
